# Algorithm for automatic analysis of electro-oculographic data

**DOI:** 10.1186/1475-925X-12-110

**Published:** 2013-10-25

**Authors:** Kati Pettersson, Sharman Jagadeesan, Kristian Lukander, Andreas Henelius, Edward Hæggström, Kiti Müller

**Affiliations:** 1 Brain Work Research Center, Finnish Institute of Occupational Health, Topeliuksenkatu 41aA, Helsinki 00250, Finland; 2 Electronics Research Laboratory, Department of Physics, University of Helsinki, Gustaf Hällströmin katu 2, P. O. Box 64, Helsinki FIN-00014, Finland

**Keywords:** EOG, Saccade, Blink, Auto-calibrating, Data mining

## Abstract

**Background:**

Large amounts of electro-oculographic (EOG) data, recorded during electroencephalographic (EEG) measurements, go underutilized. We present an automatic, auto-calibrating algorithm that allows efficient analysis of such data sets.

**Methods:**

The auto-calibration is based on automatic threshold value estimation. Amplitude threshold values for saccades and blinks are determined based on features in the recorded signal. The performance of the developed algorithm was tested by analyzing 4854 saccades and 213 blinks recorded in two different conditions: a task where the eye movements were controlled (saccade task) and a task with free viewing (multitask). The results were compared with results from a video-oculography (VOG) device and manually scored blinks.

**Results:**

The algorithm achieved 93% detection sensitivity for blinks with 4% false positive rate. The detection sensitivity for horizontal saccades was between 98% and 100%, and for oblique saccades between 95% and 100%. The classification sensitivity for horizontal and large oblique saccades (10 deg) was larger than 89%, and for vertical saccades larger than 82%. The duration and peak velocities of the detected horizontal saccades were similar to those in the literature. In the multitask measurement the detection sensitivity for saccades was 97% with a 6% false positive rate.

**Conclusion:**

The developed algorithm enables reliable analysis of EOG data recorded both during EEG and as a separate metrics.

## Background

Fatigue caused by sleep deprivation has a negative impact on human performance [1,2], thus increasing the possibility of accidents and human errors [2-4]. A reliable automatic on-line monitor of fatigue would be highly welcome e.g. in safety critical occupations [4,5]. Electro-oculography (EOG) is routinely registered by electroencephalography (EEG) setups to allow removal of eye movement artifacts [6]. Parameters derived from both EEG [7-10] and EOG [11-15] have shown to be promising indicators of fatigue. In addition, combining these parameters have given even better results [16-18]. However, large amounts of EOG data is underutilized, since the traditional EOG metrics require calibrating the relationship between recorded voltage and the corresponding eye movement. To address this issue we set out to develop a robust, automatic, auto-calibrating algorithm that classifies temporal eye parameters (saccades, blinks) from EOG data.

Usually the studies where EEG is used for monitoring subject state, subjects are driving [19,20] or navigating [21] in the simulator or they are performing cognitively demanding tasks on a computer screen [8,22]. Some studies are done in real life situations [13,23]. In these measurement setups the subjects are viewing freely and the measurement times are quite long, up to several hours. In addition, the measurement devices, setups, subjects etc. change. These measurement conditions: 1) lack of calibration 2) free viewing 3) long measurements and 4) changing measurement conditions presents challenges to EOG signal denoising and eye movement detection.

Both blink and saccade parameters, derived from EOG such as blink rate [14,16], blink duration [19,20,23], blink amplitude and eye closing time [11], saccade rate and eye activity [11,13,17], and saccade velocity parameters [11,17,24,25] have shown to be sensitive for fatigue (caused by sleepiness). Our previous study suggested that the peak velocity of horizontal saccades could be the most potential eye parameter for monitoring sleepiness [15]. Saccades are usually detected from EOG signal by the velocity threshold method [11,17]. To be able to set the velocity threshold value e.g. 15°deg/s [26,27], knowledge on the relationship between recorded voltage and eye movement is needed. Blink detection is somewhat simpler since blinks induce a larger change to the EOG signal than saccades. Thus blinks have been detected without calibration or scored visually [14,20].

Baseline drift in the EOG signal is mainly unrelated to eye movements and is caused e.g. by electrode polarization [28], or changes in contact impedance due to sweating [29]. In addition, high frequency noise is picked up from e.g. powerlines, muscle activity, and subject's movement [29]. Denoising EOG signal is challenging since eye movements are usually non-repetitive making the EOG signal unpredictable. Consequently, methods that need structural and temporal knowledge about the expected signal cannot be used. In addition, reliable eye movement classification and further analyses require undistorted edges, amplitude, and durations of the eye movements signal. Baseline drift has been removed from EOG signals by wavelet [30], and highpass [19] filters whereas lowpass [31] and median filters [30,32,34] have been used to remove high frequency noise. Unfortunately, digital filtering distorts the saccade parameters [31,33,34].

The rising trend neuroergonomics and human factors research is to carry out studies in real-life environments. For this purpose calibration free but robust analysis approaches for EOG data are needed. To our knowledge no calibration free algorithm for robust saccade and blink detection has been published.

Here we present an automatic, auto-calibrating algorithm, which estimates automatically the threshold values for saccades and blinks, allowing effective analyzes of EOG data sets. It can potentially open up a path towards using EOG in naturalistic environments.

## Methods

**Figure 1:**
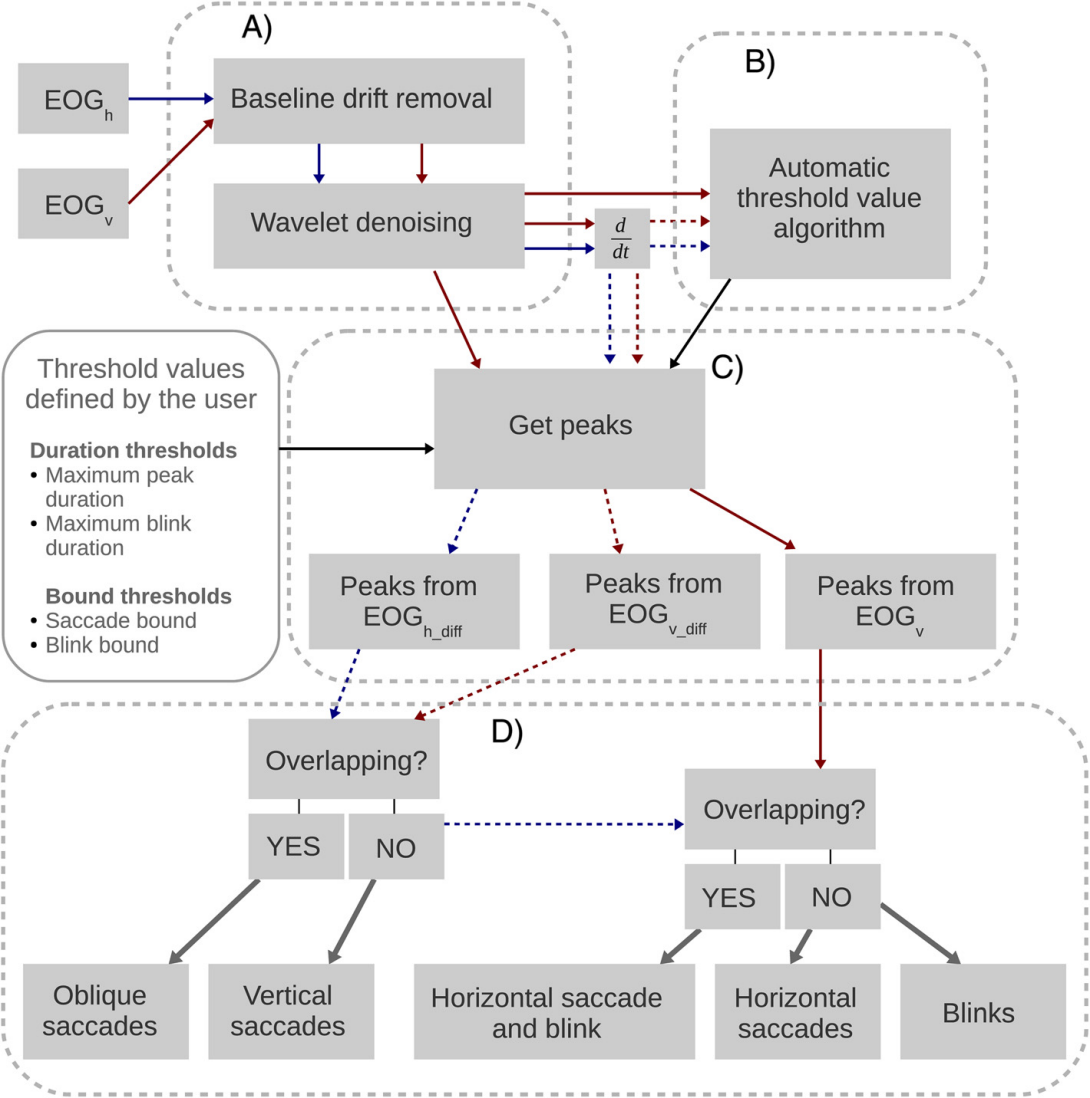
**Block diagram of the algorithm.** The algorithm consists of four blocks: **(A)** Artifact removal, **(B)** Estimation of amplitude threshold values, **(C)** Feature extraction, and **(D)** Classification. The red arrow represents EOG_v_ and the blue arrow EOG_h_ (see text for details). The time derivative of the EOG signal is a velocity signal of the eye (EOG_v_diff_ and EOG_h_diff_). The red dashed arrow is EOG_v_diff_ and the blue dashed arrow is EOG_h_diff_. The black arrows represent threshold values whereas the gray arrows point to the classification results. Maximum peak value threshold defines the maximum duration for single peak whereas the maximum blink duration threshold value defines the maximum duration for the blink. Bound threshold value defines how the start and end point of a feature (saccade or blink) is defined (see text for details).

The algorithm consists of four blocks (Figure 1): A) artifact removal, B) estimation of amplitude threshold values, C) feature extraction, and D) feature classification.

### Input signal

**Figure 2:**
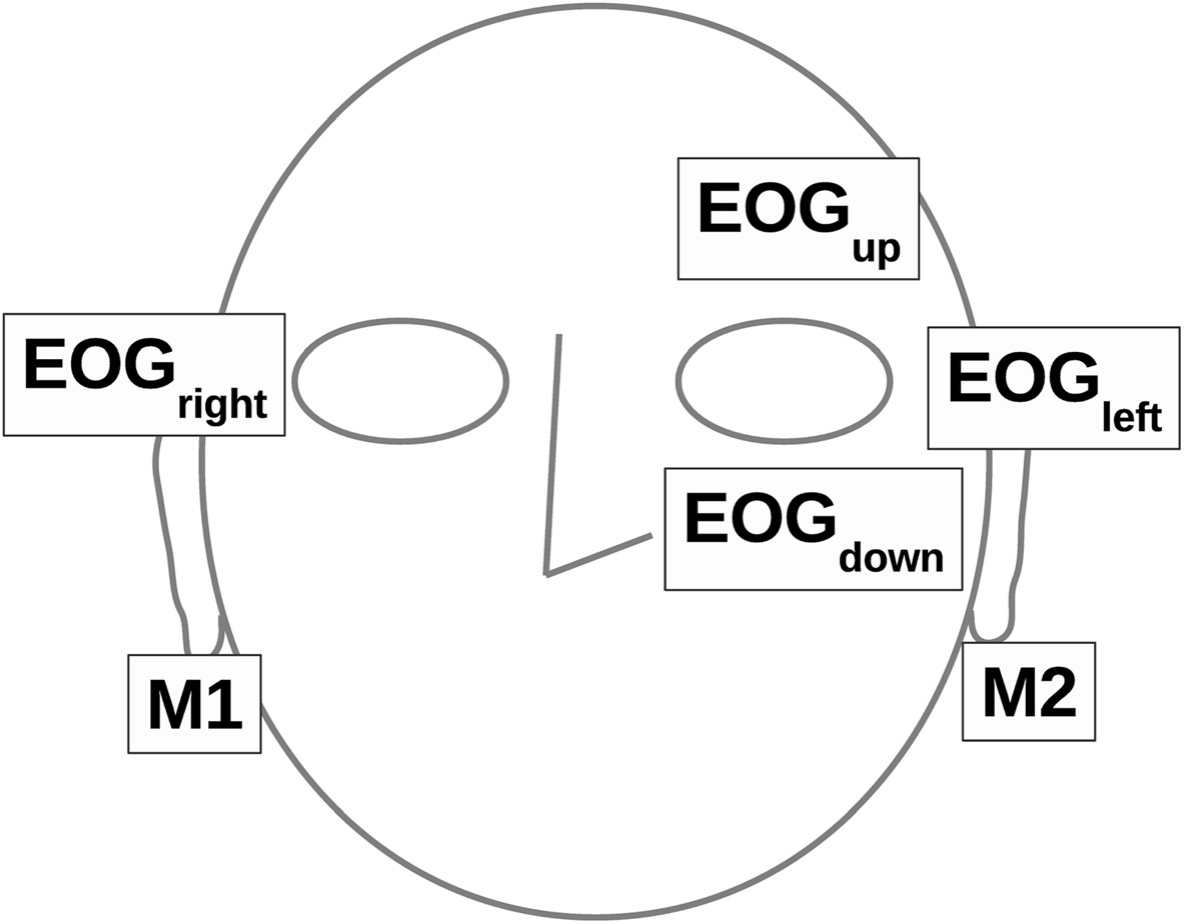
**Electrode placement.** EOG_v_ is the difference between the voltage recorded by the upper and lower eye electrode (EOG_v_ = EOG_up_ – EOG_down_) whereas EOG_h_ is the difference between the voltage recorded by the right and left eye electrodes (EOG_h_ = EOG_right_ – EOG_left_).

**Figure 3:**
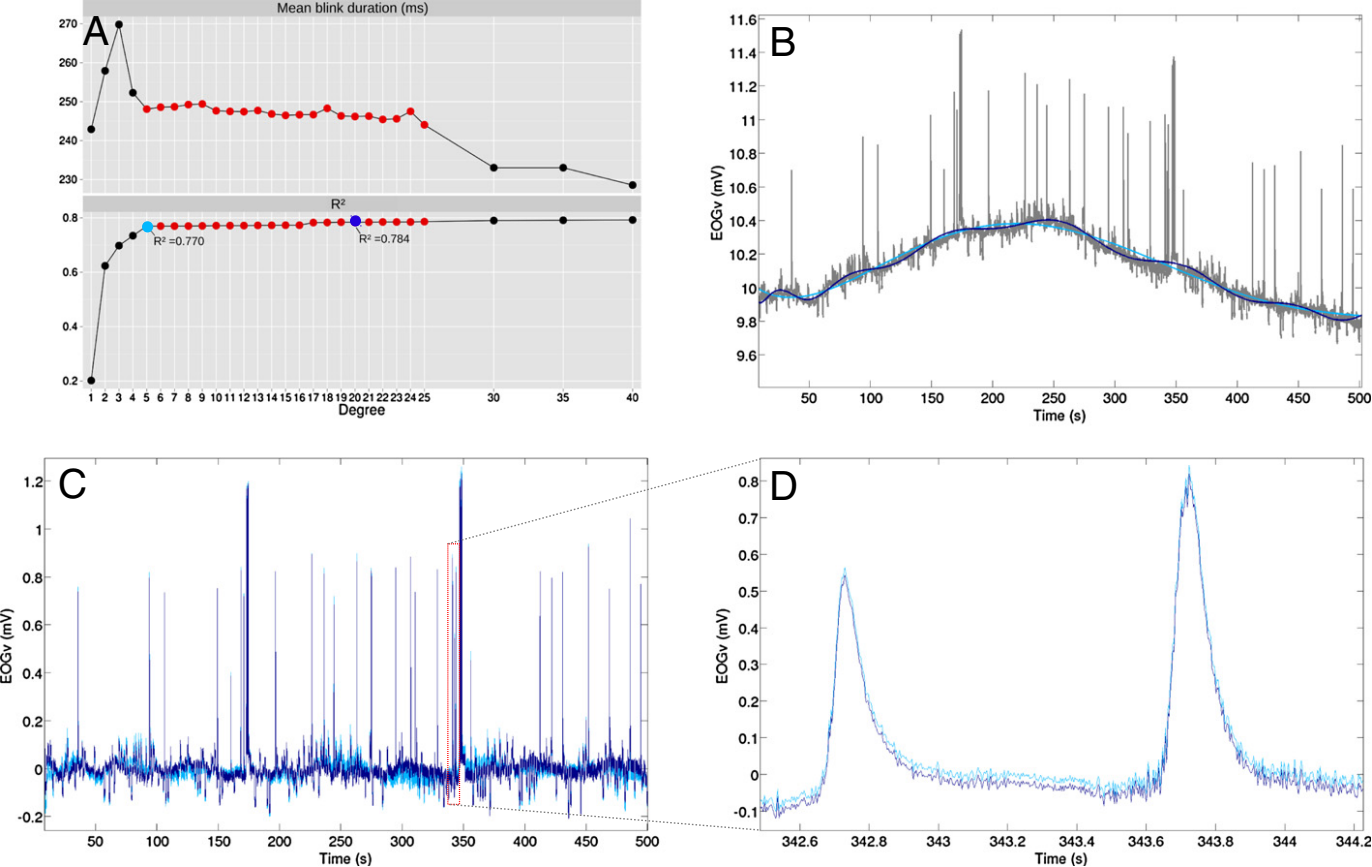
**Baseline drift removal. A)** R^2^ and mean blink duration (EOG_v_ signal from subject #1) as a function of polynomial degree. The fifth order polynomial (R^2^ = 0.770) is marked with light blue and 20th order (R^2^ = 0.784) is marked with blue. The Figure shows that the R^2^ values and the mean blink durations are quite stationary for polynomials of order 5 to 25. **B)** Fitted fifth (light blue) and 20th order (blue) polynomials are plotted on the top of the raw signal (gray). The 20th order polynomial models the signal more closely than the 5th order polynomial. However, the difference between the fit (5th and 20th order) polynomials in the baseline denoised signals **(C)** and single blinks **(D)** is rather small. This figure shows that the baseline drift removal is fairly robust.

**Figure 4:**
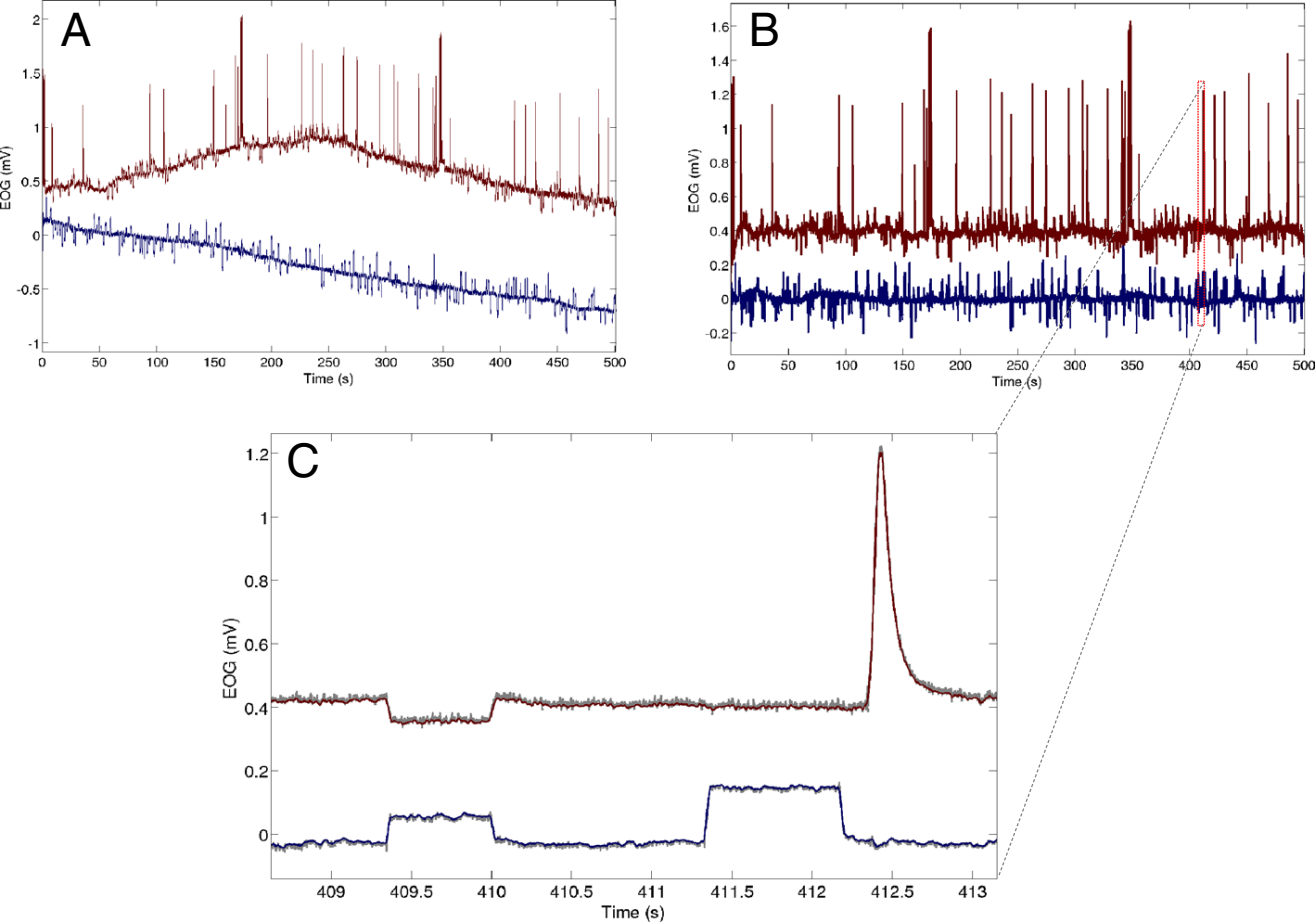
**Denoising. A)** Raw EOG signal (EOG_v_ is red and EOG_h_ is blue) and **B)** baseline drift removed EOG signals. **C)** Denoised EOG signals, the gray signal represents the EOG signal before wavelet denoising.

The EOG signal was recorded with a NeurOne amplifier (Mega Electronics Ltd., Kuopio, Finland); 500 Hz sampling rate, direct current (DC) measurement, lowpass filter (−3dB cutoff 125 Hz). The input signals, horizontal EOG (EOG_h_) and vertical EOG (EOG_v_), were measured with four AgAg-Cl electrodes (Technomed Europe, Maastricht, The Netherlands) at the outer canthi of both eyes, and above and below the left eye. The measurement was monopolar and the electrodes were referenced to the right mastoid (M1) and grounded to the left mastoid (M2). The electrode placement is presented in Figure 2. The raw EOG_h_ and EOG_v_ signals are presented in Figures 3 and 4.

### Artifact removal

The baseline drift in the horizontal and vertical EOG signals was removed by first modeling the drift by a polynomial of degree 20 from the signal, and then subtracting the polynomial from the data. A high-degree polynomial was chosen to ensure that the polynomial accurately models the baseline of the EOG signal. Possible overfitting was studied by examining R2 values, eye movement parameters, and the raw EOG signal for polynomials of varying degree (Figure 3). Noise removal by wavelet truncation was performed prior to feature extraction. The wavelet coefficients with pseudo-frequencies above 100 Hz, corresponding to fine structure (i.e. noise), were removed from the wavelet decomposed signal before reconstruction. The data retained in the coarse wavelet coefficients is the data relevant for EOG feature extraction. Haar wavelets were used because they resemble the eye movement signal and have been used to identify e.g. rapid eye movements (REM) [35], saccades and fixations [32]. Denoised EOG signals are presented in Figure 4.

### Estimation of amplitude threshold values

Saccades and blinks were identified in the EOG signal using threshold values. Auto-calibration was implemented by determining threshold values from features in the signal.

**Figure 5:**
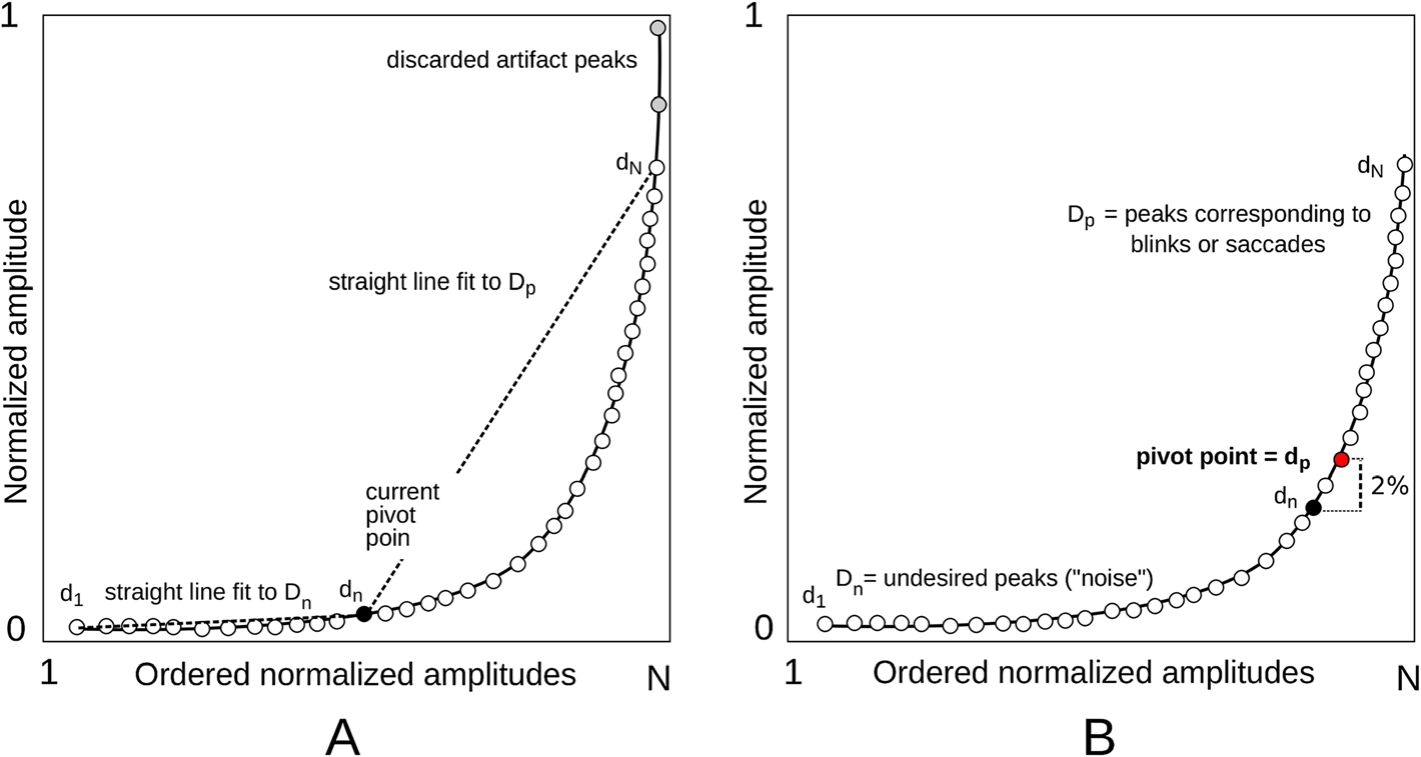
**Amplitude threshold estimation.** Schematic figure of amplitude threshold estimation. The black curve with a white circle represents the scaled peaks plotted in ascending order. **A)** The artifact peaks (gray circles) are defined as outliers and are discarded. The candidate pivot point d_n_ (black circle) is estimated by fitting two straight lines to two datasets (D_n_ and D_p_, see text for details) and by subsequently minimizing the least squares error (LSE) between the fitted lines and the curve. **B)** The pivot point (d_p_) is determined as the point with an amplitude 2% higher than that of d_n_. The point d_p_ divides the data set into noise (D_n_) and peaks corresponding to blinks or saccades (D_p_).

First, all local maxima were extracted from the absolute value of the signal. The amplitudes of these samples were scaled to the range [0, 1] and sorted in ascending order. A schematic curve of the scaled peaks is shown in Figure 5. Large artifact peaks (outliers) were removed (starting from the high-end and going towards the low-end) by discarding samples above the point where the difference between sequential peaks was less than 0.03 (curve of peaks scaled to [0, 1], Figure 5).

The set D represents all N sorted peaks after artifact removal: D = [d_1_, …, d_N_]. The points in this set were divided into two classes: D_n_ = noise peaks and D_p_ = peaks corresponding to saccades or blinks. The pivot point d_p_ dividing the data into these classes was determined as follows. D was first split into two preliminary sets D_n_ = [d_n_ … d_n_ + d_(N/2 - 1)_] and D_p_= [d_n_ + d_(N/2 - 1)_…d_N_], where dn represents the candidate pivot point. A straight line was fit to each set and the least-squares error (LSE) for each set was calculated. The candidate pivot point d_n_ was increased in steps of 1, starting from n = 10 until n = (N/2), and line fitting was done in the two new candidate sets. This process was repeated until n = N, after which the final pivot point d_p_ was determined as the point with an 2% higher amplitude than that in d_n_. This ensures that most artifacts are discarded from further analysis.

The threshold for horizontal and vertical saccades was defined in a similar fashion from the time derivative of the EOG signal (EOG_h_diff_ and EOG_v_diff_) whereas the threshold for the blinks was defined from EOG_v_.

### Feature extraction

Saccades and blinks are the features of interest in the EOG signal. Horizontal saccades were determined from the EOG_h_diff_, vertical saccades from EOG_v_diff,_ and blinks using EOG_v_diff_ and EOG_v_.

#### Peak detection

Local maxima exceeding the amplitude threshold value determined earlier were located. The start and end points for each detected peak was defined by a manually set bound value (% of local max, e.g. 10%). If the left boundary was found, the algorithm continued to search for the right boundary until: 1) the signal level corresponded to the bound value, 2) the signal level exceeded the peak value, or 3) a limit determined by the maximum peak duration was exceeded. If both boundaries were found the peak was stored.

**Figure 6:**
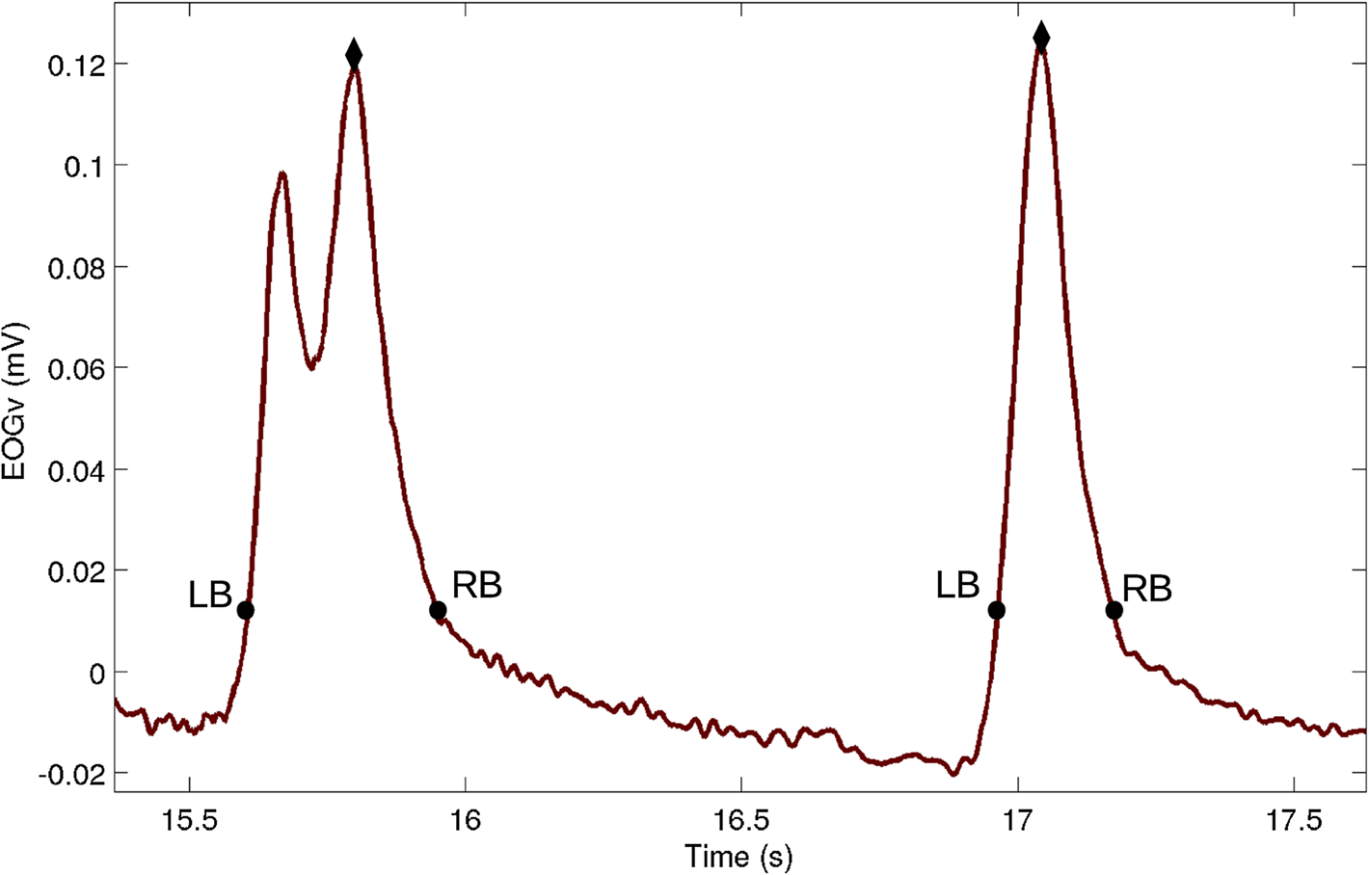
**Double-peaked blink.** Example of a double-peaked (left) and normal blink (right). The start of the blink is marked LB (left bound) whereas the end of the blink is marked RB (right bound). The peak is marked with a black diamond.

The described peak detection algorithm can cope with double-peaked blinks (Figure 6), that occur when the subject blinks twice in a short period of time.

#### Extraction of saccades and blinks

The methods for finding saccades and blinks were partially similar. Both methods start with peak extraction. Peaks were extracted from EOG_h_diff_ and EOG_v_diff_ by searching (both positive and negative) peaks exceeding the amplitude threshold value determined earlier. These extracted peaks are saccade candidates. However, the blinks and saccades need to be separated from peaks detected in EOG_v_diff_. If an up-and-down pattern was found in EOG_v_diff_ inside a given time window (maximum blink duration), the corresponding up-peak in the EOG_v_ was searched for. If the found up-peak was larger than the determined blink amplitude threshold value, the vertical saccade candidate was discarded and the peak was marked as a blink.

### Classification

After the features of interest were extracted from both the horizontal and vertical signals, they were classified. Horizontal and vertical saccades were extracted from the EOG_h_diff_ and EOG_v_diff_, respectively. An oblique saccade was found when a horizontal and a vertical saccade occurred simultaneously. The fourth category defined a blink whereas the fifth one described a situation where a horizontal saccade and a blink coexisted.

### Output parameters

The output contains 1) number of features, 2) duration of features, 3) peak velocity (V/sec) for saccades, 4) eye acceleration and deceleration during saccades, and 5) eye closing and opening times for blinks.

### Algorithm performance

#### Algorithm implementation

The algorithm was implemented in Matlab (Matlab R2012a, The MathWorks Inc., Natick, Massachusetts). A wavelet based artifact removal was applied using the Wavelab850-package [36]. The threshold value for ‘saccade’ and ‘blink’ bound was set to 10% of the amplitude peak. The maximum peak duration threshold was set to 0.80 sec and the maximum blink duration was set to 1.2 sec. These values were chosen after testing different values during the development process. During the development process we tested the proposed parameters on different kinds of datasets (e.g. Sallinen et al. [22], Gould et al. [37], Hirvonen et al. [15]).

#### Subjects

Three laboratory personnel (two male and one female) volunteered for this study. The subjects were 26, 32, and 33 years old. They all had normal vision and did not report any health problems. The subjects were informed about the objectives and conditions of the study. This study complied with the Helsinki Declaration and was approved by the ethics committee of the Hospital District of Helsinki and Uusimaa, Finland.

#### Tasks

**Figure 7:**
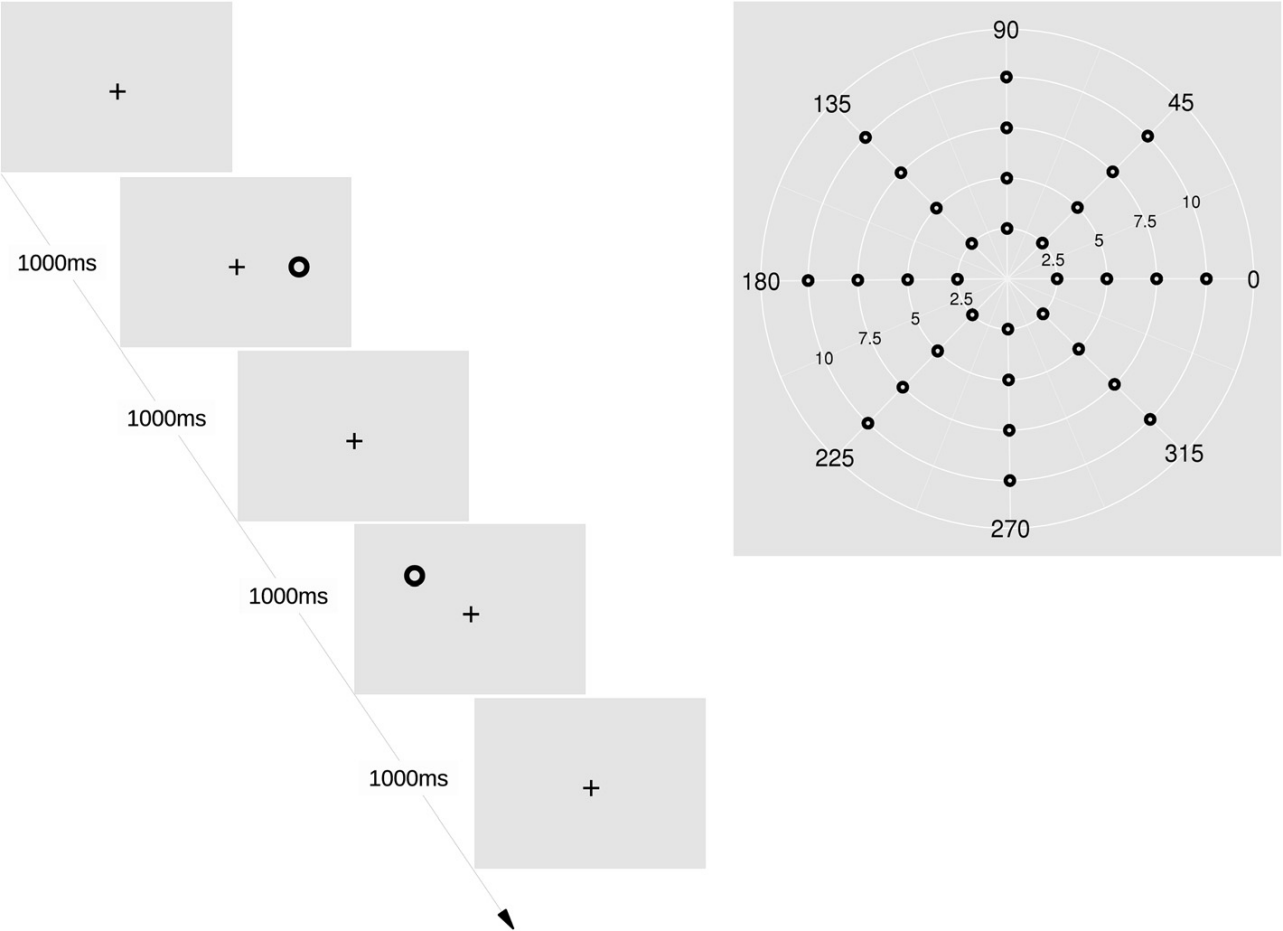
**Ring saccade task.** Ring saccade task sequence (left). The cross was used as a central fixation point and a donut as a target stimulus. Target positions with 8 directions and 4 amplitudes (2.5, 5, 7.5, and 10 deg of visual angle) are presented on the right.

The performance of the algorithm was tested using two different kinds of tasks to estimate the robustness (sensitivity, specificity) and consistency^a^ of the algorithm. In experiment 1 the subjects performed a ring saccade task where the eye movements were controlled. With this data we were able to assess how the algorithm detects and classifies the saccades with different amplitudes and directions. The ring saccade task (Figure 7) task was implemented using a custom in-house developed Windows application designed for stimulus presentation. The subject made 640 saccades along eight directions (0, 45, 90, 135, 180, 225, 270, 315 degrees) with four different saccade amplitudes (2.5, 5, 7.5, and 10 degrees of visual angle). Saccades to each position (see Figure 7) were repeated twenty times in a randomized manner. The task was divided into four 160 saccade trials, each trial lasting 168 sec. There was a 5 sec pause between each trial, during which the subjects were instructed to blink repeatedly to lessen the need to blink during the trial. The task lasted 11.5 minutes. The subjects were instructed to look at the location of a central fixation point until the target stimulus appeared after which they should move their gaze as quickly as possible to the target stimulus. When the stimulus disappeared, they were instructed to move their gaze back to the central fixation point (return saccade). Both saccades to stimuli and return saccades were used in the analyzes. The reaction times to the stimulus was not analyzed.

**Figure 8:**
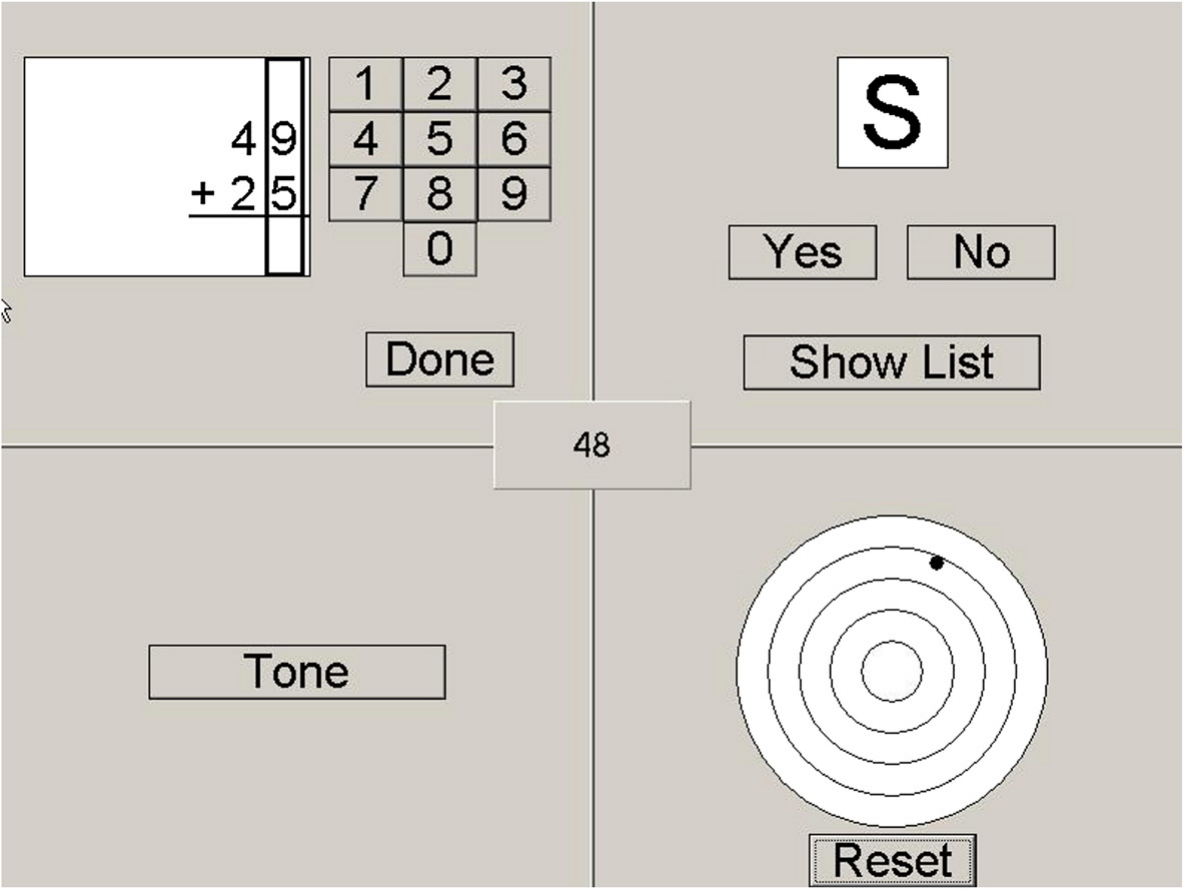
**Brain@Work multitask.** The Brain@Work multitask consists of four simultaneously functioning subtasks; arithmetic, short term memory, auditory and visual vigilance subtasks [22].

In experiment 2 the Brain@Work multitask [22] was used to test the performance of the algorithm in a task where the eye movements are not controlled. The Brain@Work multitask consists of four simultaneously performed subtasks; arithmetic, short term memory, auditory and visual vigilance subtasks (see [22] for a more detailed description). The layout of the Brain@Work multitask is presented in Figure 8. The multitask lasted 5 minutes and during that time the subjects were instructed to perform the task as well as possible. All the subject's were familiar with the task and had performed it multiple times before the measurement session. Performance in the multitask was not analyzed.

Both experiments were carried out in a silent laboratory where the only light-source was the computer screen. The subjects sat in a chair 57 cm from the computer screen. A head rest was used to avoid head movements during the measurements. Subjects were also instructed to sit still and avoid blinks during actual tasks.

#### Eye movement measurements

Eye movements were recorded with EOG and VOG device. The EOG signal was used as a test signal for the algorithm and the VOG data was measured to get estimation on subjects eye movements in order to be able to study the robustness of the algorithm.

The EOG signal was measured as described earlier. We used the EyeLink (SensoMotor Instruments GmbH., Teltow, Germany) with 250 Hz sampling rate to record the VOG data. The EyeLink was calibrated with a 9-point calibration before experiments for each subject. The saccades were obtained from the EyeLink output file. The EyeLink uses a velocity/acceleration threshold method for detecting saccades and the default values for acceleration threshold (9500°/s^2^) and velocity threshold (30°/s) were used.

#### Analysis

The eye tracker data, EOG signal, and tasks were synchronized with 5 blinks which each subject made in the beginning and end of each task.

In the experiment 1, the ring saccade task sequence, EyeLink saccade detection and EOG saccades were compared. If the saccade, which corresponded to the stimulus was found from both the EyeLink and EOG signal, the saccade was marked as ‘true positive’. If there was no saccade detection in the EOG data but EyeLink data indicated that the subject had made the saccade corresponding to the stimulus, the saccade was marked as ‘false negative’. If there was no saccade detection in both EyeLink and EOG data for a presented saccade stimulus we assumed that the subject had not made the saccade to the stimulus direction and the saccade was marked as ‘true negative’.

The sensitivity of the saccade classification was estimated by using true positive detections by comparing the EOG classification to the task stimulus direction: If a true positive saccade was classified correctly compared to the stimulus direction, it was marked as ‘true positive’. Correspondingly a incorrectly classified saccade was marked as ‘false negative’. A false positive estimator was not available in this experiment.

Mean durations and mean peak velocities of correctly classified horizontal saccades are presented to study the influence of denoising. To allow the presentation of peak velocity values in commonly used units (deg/s), four random 10-degree horizontal saccades were used to define the relationship between voltage and visual angle. This was done separately for each subject. The peak velocity value for a certain horizontal saccade was the peak value of the EOG_h_diff_ signal.

The number of blinks was visually scored from the vertical EOG signal according to the AASM Manual for the Scoring of Sleep and Associated Events [38]. The results of the manual scoring and those obtained by the algorithm were compared.

In experiment 2, the saccades collected with EyeLink and with EOG were compared to get an estimation on the number of false positive detections. If a saccade was found with both methods it was marked as ‘true positive’. If a saccade was only detected in the EOG data, the saccade was marked as ‘false positive’, while a saccade was marked as ‘false negative’ if only the EyeLink found the saccade. A true negative estimator was not available in this experiment.

## Results

### Experiment 1

**Figure 9:**
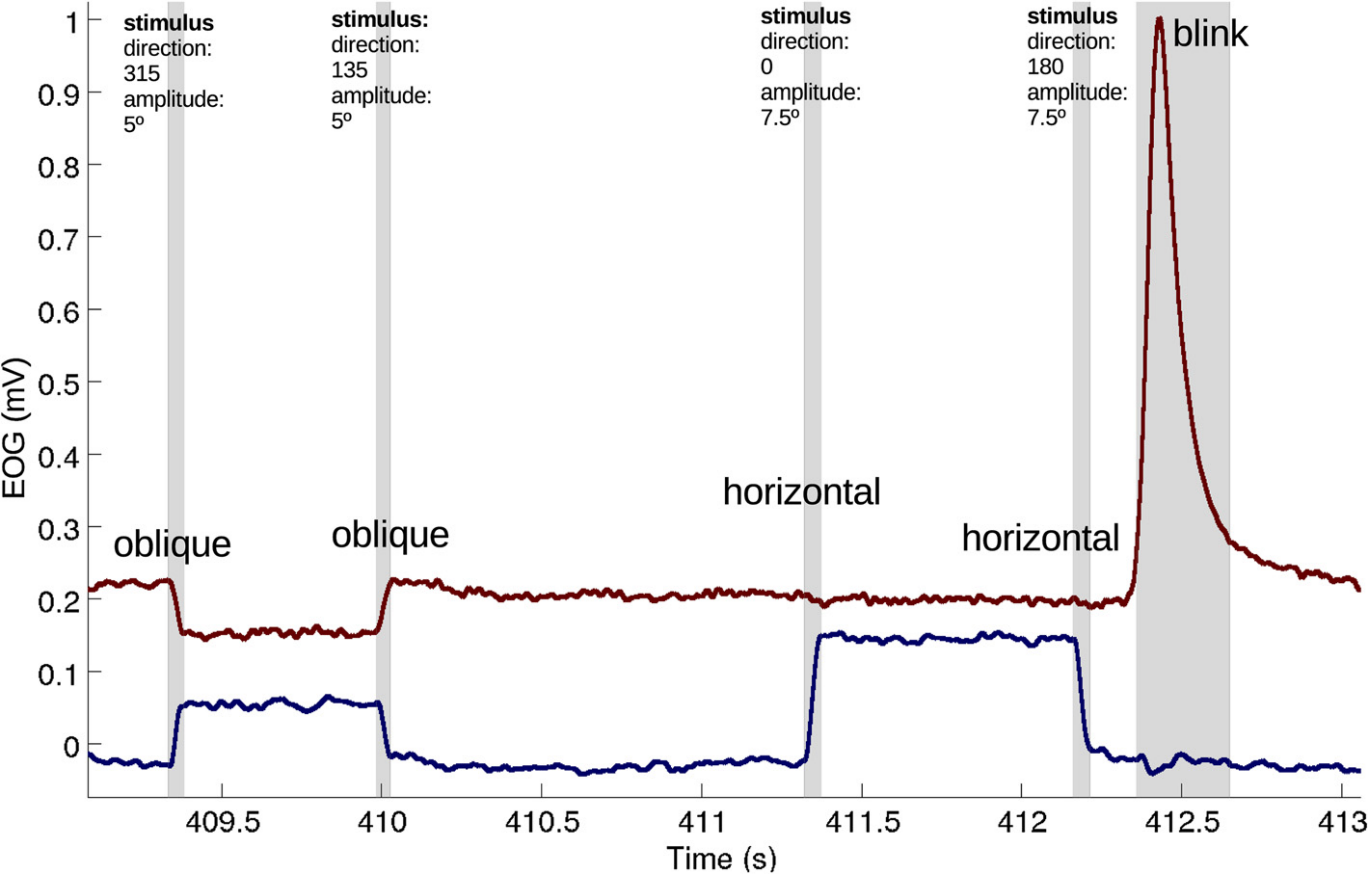
**Automatically classified EOG signal.** EOG_v_ (red) and the blue signal is EOG_h_ (blue). Stimulus information is presented above the eye movement, classified saccades and blinks are marked gray.

**Figure 10:**
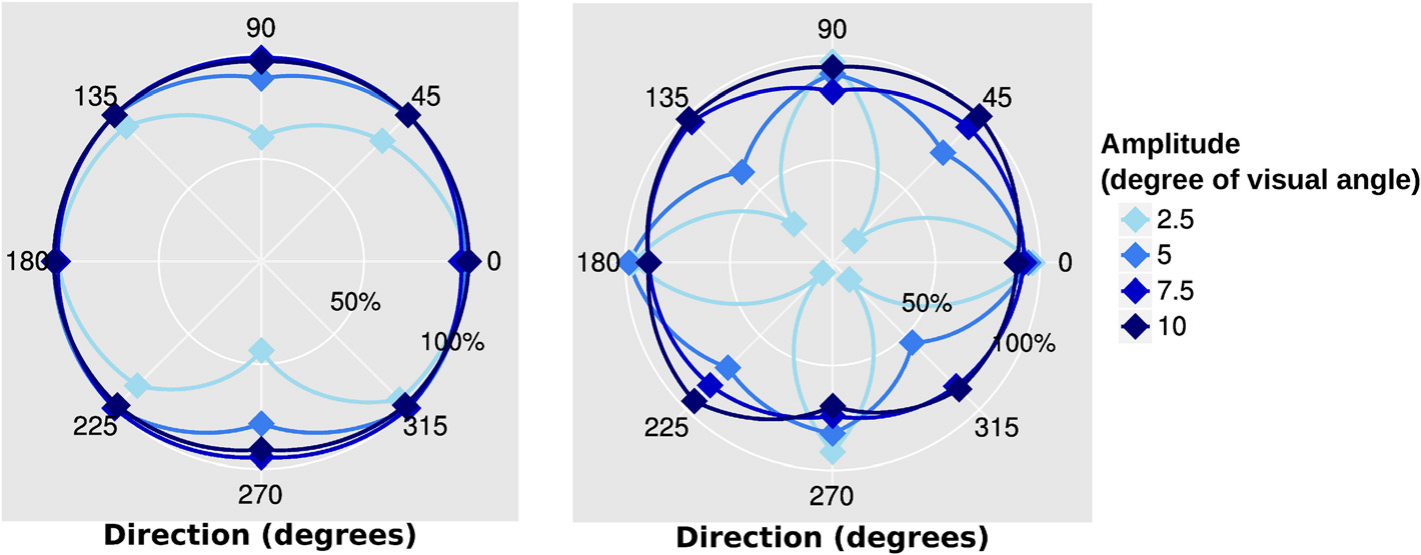
**Detection and classification.** Fractions of found saccades (left) and fractions of correctly classified saccades (right) along all 8 directions.

We analyzed 1920 saccades, of which 231 saccades were removed from the final analysis because of clear artifacts from subject movement (e.g. coughing). An example of automatically classified signals is presented in Figure 9 and the detection and classification sensitivity values of all saccades are presented in Table 1 and Figure 10. The sensitivity for horizontal saccade detection was between 98% and 100% (for all amplitudes). Most horizontal saccades were classified correctly as horizontal (89%-96%). For vertical saccades with an amplitude of 7.5 or 10 deg, the detection sensitivity was higher than 94%. However, the detection sensitivity for 2.5 deg saccades was 54% and for 5 deg saccades 87%. The classification performance with vertical saccades was between 78%-95%. Some vertical movements (≤22%) were classified as oblique. The detection sensitivity for oblique saccades was between 91% and 100%, for all amplitudes. However, the classification of the small oblique saccades was challenging since most of them were classified as horizontal ones. Whereas the large ones were often classified as oblique movements. The peak velocities and the durations of horizontal saccades are presented in Table 2.

**Table 1:** Detection and classification performance

**Saccade type**	**Amplitude (deg)**	**Stimulus N**	**Sensitivity for saccade detection (%)**	**Sensitivity for saccade classification (%)**
Horizontal	2.5	112	99	96
	5	108	100	96
	7.5	108	98	90
	10	107	100	89
Vertical	2.5	106	54	95
	5	102	87	87
	7.5	106	98	78
	10	108	94	82
Oblique	2.5	196	91	16
	5	212	100	66
	7.5	212	100	89
	10	212	100	95

**Table 2:** Mean duration and peak velocity of correctly classified horizontal saccades

**Amplitude (deg)**	**Mean duration (ms)**	**Mean peak velocity (deg/s)**
2.5	37 ± 13	232 ± 72
5	45 ± 10	333 ± 75
7.5	51 ± 7	412 ± 61
10	55 ± 12	478 ± 66
2.5	37 ± 13	232 ± 72

The number of visually scored blinks was 213. The algorithm's detection sensitivity for blinks was 93% whereas the false positive rate (incorrectly classified blinks) was 4%. The mean duration of the correctly detected blinks was 257 ± 103 ms.

### Experiment 2

Altogether, EyeLink found 2895 saccades from the data collected during the multitask measurement. The sensitivity for the EOG measurement was 74% and the false positive rate was 27%. However, when only the saccades with durations between 30ms and 80ms were used, the EyeLink detected 1351 saccades and the algorithm's sensitivity for saccade detection was 97% and the false positive rate 6%.

## Discussion

The purpose of this study was to develop an automatic, auto-calibrating algorithm enabling analysis of eye movement parameters from routine EEG and EOG measurements. To our knowledge such a calibration free algorithm for both saccade and blink detection has not yet been published. The auto-calibration is based on automatic threshold value estimation. Amplitude threshold values for saccades and blinks are determined based on features in the recorded signal. The presented algorithm allows the use of the velocity threshold method without calibration. The performance of the algorithm was tested with EOG data collected from three subjects in a ring saccade task and 5 minute multitask measurement. The EOG data was compared to VOG device data in order to get an estimation on subject's eye movements. Altogether 1689 saccades and 213 visually scored blinks from the ring saccade task and 2895 saccades from the multitask measurement were analyzed. While data from only three subjects was used, the total number of recorded saccades was large enough to test the robustness and consistency of the algorithm.

### Robustness and consistency

The robustness and consistency of the algorithm was tested by measuring EOG signal in two separate experiments and the results were compared with VOG data which was measured simultaneously. The measurement setup was implemented with the aim of minimizing known sources of artifacts. Subject motion was restricted with a head rest. We used a signal recorded from a human subject since such data includes artifacts encountered in routine measurements such as noise from muscle activity, different physiology, and differences in electrode placement between subjects.

In experiment 1 the number of true positive detections was high for horizontal and oblique saccades resulting in a sensitivity value larger than 91%. In addition, large vertical saccades (7.5 and 10 deg) were found with a detection sensitivity exceeding 94%. However, small vertical saccades (2.5 and 5 deg) were hard to detect (54% and 87% sensitivity, respectively). This result was expected since the vertical components of the EOG signal are weaker than the horizontal components and since eyelid movements induce a major artifact in the vertical EOG signal [39].

Classification sensitivity for horizontal saccades was 89% - 96% whereas fewer vertical saccades were classified correctly (78% - 95%). However, some horizontal (≤11%) and vertical movements (≤22%) were classified as oblique saccades since the saccade introduced a component into both the horizontal and vertical signals. As the phenomenon is more common with larger saccades (7.5 and 10 deg) we believe that this could be the result of a slight head tilt of the subject. Most of the small (2.5 deg) oblique saccades were classified as horizontal ones in a resulting classification sensitivity of only 16%, probably because the vertical components are weaker than the horizontal components (see previous chapter). In contrast, most (95%) large saccades (10 deg) were correctly classified as oblique. The detection rate sensitivity for blinks was 93%, with a 4% false positive rate.

Experiment 2 gave us the opportunity to investigate the number of false positive detections compared to VOG data. When all saccades were used the sensitivity of the algorithm was 74% and false positive rate was relatively high (27%). Most of the false positives were artifact peaks. The duration of these peaks was less than 25 ms whereas most of the missed saccades were small lasted less than 20 ms. In addition, experiment 1 demonstrated that the detection sensitivity for smaller saccades is less than for large ones. Based on these findings we decided to rule out smaller than 5 deg saccades from the analysis by using a saccade minimum duration of 30 ms [27]. Since the main sequence is linear up to 15 – 20 deg saccades [40] and most of the saccades are smaller than 15 deg [41] we used 80 ms the maximum duration for saccade. When only the saccades with durations between 30 ms and 80 ms were used, the sensitivity of the algorithm increased to 97% and the number of false positive detections decreased to 6%.

Based on the results from a large number of saccades (4584) and blinks (213), the consistency of the algorithm performance is good. We were not able to estimate the specificity value for the algorithm since the lack of a false positive estimator in experiment 1 and a true negative estimator in experiment 2.

Even though the automatically estimated threshold value for each subject and eye movement (saccade, blink) is used to avoid selection bias, the algorithm detects some false positive peaks. For blinks, the false positive rate was 4%. Most of the (88%) falsely detected blinks lasted less than 90 ms. This implies that we should add a minimum duration threshold value for blinks to improve the sensitivity of the algorithm. In addition, experiment 2 demonstrated that by using only saccades with durations between 30 ms and 80 ms the detection sensitivity increased from 74% to 97% and the false positive rate decreased from 27% to 6%.

The presented estimations on robustness and consistency are strongly dependent on the measurement result of the used VOG device (EyeLink). Further research are needed to get more elaborate knowledge on the robustness and consistency of the algorithm.

### Denoising

Robust baseline drift removal has a significant effect on the performance of the feature extraction depends. Drift removal was implemented using a 20 degree polynomial to model the baseline drift of the EOG signal. There is a risk of overfitting the EOG signal by using such a high degree polynomial. Figure 3. shows that a 5 degree polynomial could suffice. Alternatively, adaptive baseline-drift removal could be carried out by first estimating the degree of baseline drift present. This has not yet been investigated.

Removing high frequency noise from an EOG signal is challenging. Traditional lowpass filters distort eye movement parameters [31,33]. However, median filtering has been proposed to be an effective tool for denoising EOG signals [32,34]. During the development process we tested median filtering. To get high detection rates for the eye movements, the window of the median filter had to be so large that the eye movement parameters were distorted. This was due to the characteristics of our peak detection algorithm and the noisy EOG signal. Hence, wavelet denoising was more suitable for the developed peak detection algorithm.

Horizontal saccade durations, peak velocity values and blink durations are reported to show that the denoising does not influence the temporal parameters of the signal. The recorded saccade durations are similar to those presented by Bahill and colleagues (1981) in their normative data; their 5 deg horizontal saccades lasted 42 ± 8 ms whereas the 10 deg ones lasted 51 ± 8 ms [27]. This is comparable to our results: 45 ± 10 ms and 55 ± 12 ms, respectively.

The measured peak velocity values for 5 deg (333 ± 75 deg/s) and 10 deg saccades (478 ± 66 deg/s) are higher than in Bahill et al. [27] who reported on 5 deg (261 ± 42 deg/s) and for 10 deg (410 ± 67 deg/s) saccades. The difference between the values can be a result of using different measurement techniques and data analysis methods [42], inter- and intrasubject variability [43] as well as the frequency of the visual stimulus sequence [44]. However, our denoising method does not decrease the peak velocity values as reported for traditional digital filters [31].

The mean blink duration was 257 ± 103 ms. The blink duration varies depending on its origin (spontaneous/endogenous or voluntary) and on the measurement technique [45,46]. Stern et al. [46] concluded that the durations of endogenous blinks are 250 ms-1000 ms whereas Caffier et al. [45] reported blink durations of 202(±6)ms measured with infrared-oculography. Our blink durations are similar to those reported in previous studies.

However, they are short which may be due to differences between measurement techniques and origin of the blinks. In our data most blinks were voluntary since the subjects were instructed to blink repeatedly during pauses and to avoid blinking during the actual task.

### Usability

Here we present the 1st version of the algorithm. The algorithm is not optimized for speed (analysis time with a laptop^b^: 1.6sec data/sec or 3.23 eye parameters/sec), but it is a robust tool for detecting blink and horizontal saccades. The algorithm is not yet ready for clinical work, but we believe that the fact that it does not distort the saccade parameters may facilitate inter-lab round robin testing and validation. Finally, the fact that the algorithm was successfully used for blink detection by a colleague (on a different data set) who did not partake in the development is an indication of its usability.

### Summary

Based on our results the developed algorithm reliably detects blinks and horizontal saccades, and reliably classifies horizontal saccades. These two eye parameters have been the most studied ones in different areas of neurophysiology. For instance, saccadic eye movements have been used widely in fatigue and sleepiness research [13,15,17,25], and in studies of psychological and cognitive disorders (e.g. Alzheimer's disease [47], schizophrenia [48], and ADHD [49]). Blink parameters have been reported to be sensitive to sleepiness [11,14,16,19,20] and to mental workload [50-52].

## Conclusion

We developed an algorithm enabling reliable analyses of routine EOG measurements. The algorithm offers an economic option to attain comprehensive knowledge on human cognitive performance, sleepiness, fatigue, and neurophysiology in general. Moreover, it potentially opens up a path towards enabling flexible and robust data analysis in future development of unobtrusive EOG metrics for on-line and mobile measurements.

## Endnotes

^a^We define the consistency of the algorithm as its ability to detect and classify a certain kind of event (e.g. 10 deg right saccade) across subjects and instance.

^b^Linux kernel 3.2.0-4-amd64, 3.2GiB RAM, Intel Core2 Duo 9600 2.8 GHz CPU.

## Abbreviations

EOG: Electro-oculography; EEG: Electroencephalograhy; VOG: Video-oculography; EOGh: Horizontal EOG signal; EOGv: Vertical EOG signal; EOGv_diff: Time derivate of the vertical EOG signal; EOGh_diff: Time derivate of the horizontal EOG signal; REM: Rapid eye movements; LSE: Least-squares error; DC: Direct current; ADHD: Attention deficit hyperactivity disorder.

## Competing interests

The authors declare that they have no competing interests.

## Authors’ contributions

KP: Designed and conducted the study, made data analyses and wrote the manuscript. SJ: Algorithm design and implementation (architecture). AH: Algorithm design and implementation (signal denoising), reviewed the manuscript for scientific content. KL: Gave assistance on test measurement design and execution, guidance in data analysis and interpretation, reviewed the manuscript for scientific content. EH and KM: Guidance in interpretation of the results, supervised the writing work, and reviewed the manuscript for scientific content. All authors read and approved the final manuscript.
